# Poetical Contribution

**Published:** 1847-04

**Authors:** 


*Poetical Contribution.*—Medicine is generally considered to be rather a prosaic calling; yet, if our memory of mythology serves us, Apollo was the god of healing and of the Lyre. Physicians have occasionally strayed into the walks of Poesy, and forgotten, for a season, the odor of physic among the fragrant flowers of Parnassus; returning, too, with laurels on their brows. Much might be said to disprove the idea of incompatibility in the two pursuits.—We could mention names in our own country which would suffice for its refutation—witness, Percival, Holmes, Coates, and others. Doctors, at all events, are somewhat prone to be sentimental, and we doubt not there is a world of *unwritten poetry* in the hearts of the medical fraternity. Doctors, also, it is to be considered, are proverbially given to equestrian exexcises; they ride horses, and hobbies, and why not, for the sake of variety, sometimes get astride of Pegasus! We make no farther apology than is afforded by these rambling reflections, for inserting the following effusions from a correspondent, who has some inclination for the Muses. If he should succeed in making *dry bones* live in immortal verse, he will assuredly prove himself a successful poetical practitioner—but the ***prognosis*** of the case we leave with our readers.

*For the Buffalo Medical Journal.*

Having been engaged in framing an artificial (wired) skeleton, a labor, Mr. Editor, as you are aware, which eats into one's leisure hours, the labor and time devoted naturally opened the flood-gates to thought; a few have been arrested and framed into a "rhymed lesson" which I would have subjected to ***retort*** or ***furnace*** as may be judged by you most fitting.

### TO A SKELETON.

*Tu docet, memento mori, etc.*

So teachest—unseemly to look upon,
Disrob'd of lips, eyes, heart and fleshly tongue,
Thou, with curiously wrought limb and trunk:
With cavernous orbit, graved and sunk
In a now deserted, tenantless dome,
Wherein once spirit reigned—had its home—
Invisible, to the outward seeming,
While in a world of shadows—land of dreaming.

Thus bereft, yet even now thou speakest
To whomsoever of thee yet seeketh,
Asketh, who thou wert?—what thou hast been?
If on earth's stage thou didst move a quean?
Or wert in "chivalrous south" deemed a thing—
Chattel personal?
To such questioning,
If versed in lore of anatomical schools,
And skilled in appliance of their rules,
This, thou teachest:—thine is a female mould,
Misshapen, witnessing thou hast been sold—
To fashion!—as, on thy form is trac'd
Not mark of whips,—though oft thou wert "laced"—
And, if formed, as of mother Eve 'tis book'd,
Also, ***how strangely good ribs can be crooked!***
This, this, thou teachest to the outward eye,
To inward, this, "remember thou must die!"

Thou mayest have been on earth a mother:—
Thou mayest have had a sister, brother:—
*Kindred in spirit* surely thou hadst,
Who for thee, or thou for them, did as badst;
Governing, or governed, thus for rule
Had *you* subjects, or *they* subjected tool!

A parent thou art!—to deed and thought!
And wondrous marvels thus have wrought
By such progeny, as they onward speed
Through Time's changes—giving little heed
To Eternity!—speeding their work ever,
For weal or for woe, untiring never,
Begetting saints, or legion devils,
As of virtue or of sin their travails!
These, thy mementos, never, never die,
But teach on, now, forever, and for aye,
Whatsoever we do, or think, or breathe,
Acts, thoughts, words, are not lost, but ever live! W. T.

*Buffalo, N. Y.*

